# Psychological Problems Derived from Mastectomy: A Qualitative Study

**DOI:** 10.1155/2011/132461

**Published:** 2011-06-04

**Authors:** José Manuel García Arroyo, María Luisa Domínguez López

**Affiliations:** ^1^Department of Psychiatry, Faculty of Medicine, University of Seville, 41004 Seville, Spain; ^2^Mental Health Center, 21700 La Palma del Condado, Huelva, Spain

## Abstract

Advances in treatment of breast cancer have not avoided using mastectomy in all cases, and when this happens, we are dealing with a woman who is suffering from psychological problems. In order to study this issue we have carried out a research with the collaboration of The Andalusian Association of Women with Mastectomies (AMAMA) in Seville, which provided us with a sample of 46 mastectomized women. The objective of this study is to analyze in depth the psychological reaction of women to mastectomy through its different stages from diagnosis to surgical treatment. We chose a cualitative method so as to explore the subjective components of psicologycal respons. As a result, we found in studied women: (a) The “fracture” of the “corporal imaginary” related to the disappearance of a valuable organ, linked to the feeling of loss of personal attractiveness, low self-esteem and avoidance of social relationships. (b) The problem with “femininity” has been linked to the issue of “desirability”, something innate in the “feminine position”. (c) Many of them keep in mind the idea of mutilation, as a “hole” which is impossible to integrate. (d) Finally, we demonstrate how certain features of personality made them especially vulnerable to the explained phenomena.

## 1. Introduction

Breast cancer is the most common malignant tumour in women, but nowadays there has been a great progress in its early detection and treatment, improving the diagnosis of the disease [1]. In this sense, the practice of the conservative surgery tends to be done, and it contradicts the defending ideas which asserted that “we had to remove as much as we could.” Consequently, there has been a reduction in the amount of these traumatic operations which took place with the only purpose of saving lives.

Despite these technical advances, mastectomy continues to be practiced, and several authors assert [2] that there is 40% of breast cancer cases in which it is still done. This is due to various reasons (size or position of the tumour, anticipating a bad cosmetic result, small breast, multifocal tumour, a woman's request, etc.). Although this operation may be done, sometimes an immediate reconstructive surgery of the breast is performed. This technique, becoming more and more popular in recent years [2, 3], tries to preserve the breast with its natural appearance in affected women. In order to achieve that, some resources and coordination in medical teams are necessary, what unfortunately is not always the case.

We sometimes witness situations in which these favourable conditions are not given, and we are in the presence of a woman with a lack of her breast who shows psychological disorders that can be noticeable [4–11]. Barcia [12] stated that “mastectomy causes more trauma than the cancer illness itself,” hence the need to deal in depth with this issue in order to promote a reasonable psychotherapeutic treatment for this women [13].

In this study, we intend to analyze the psychological issues affecting a mastectomised woman, particularly those due to the lack of her breast and how the subjectivity of this woman assimilates this event, involving various aspects (aesthetic, related to femininity, relational, etc.), since the breast is not just a gland that receives hormonal influences, but an area that has caught a lot of attention in our culture for being part of the “female body image” and has an indisputable erogenous value to both the affected person and her couple.

We next lay out the difficulties of the current study.

- It seems frivolous to deal with this issue in the presence of such a serious disease, where the most important thing is to save a life. Hitherto, this has been the only concern of the doctors, who have shown little interest in the matter of the feelings of women.
- Such study should include several psychological components somehow uncomfortable to talk about, such as the eroticism in women, femininity, the mechanisms of male-female attraction, and autoeroticism.
- New conceptual tools are needed because psychology and traditional psychopathology focus mainly on the conscious aspects (or cognitive), and they lack elements to approach this kind of experiences.

Here we also develop a previous research in which we coined the term “Sexualised Body Schema” (SBS) [14, 15] in 1991, in an intend to give an account of a series of strictly feminine reactions.

## 2. Material and Methods

The data of the research comes from a sample of 46 mastectomised women belonging to Andalusian Association of Mastectomised Women (AMAMA). Ethics approval was obtained from the institution, and they participated voluntarily. They were middle-aged, middle class, and both working and nonworking women. The researchers performance took place in the doctor's office at the Department of Psychiatry of the Faculty of Medicine in Seville. Interviewers were all doctors.

The method consists in a series of scheduled interviews in which the role of the interviewer is to facilitate the free speech in women making possible the unrestricted communication of their illness experience at its different stages. To achieve this aim the researchers have to listen and write down carefully whatever the woman says, and anything that may disturb or misunderstand the compilation of these statements must be put aside (psychological or psychiatric theories, personal beliefs, worries, allusions to the body, value judgments, etc.). By doing so, we get a spontaneous verbal material not influenced by the observer, and, as a result, the recorded sentences come to be isomorphic with what happens in woman's “inner” when they express the experienced distress regarding their breast operation.

The analysis we propose is complex because of the multiple psychological components of these experiences in which each woman discovers a particular vision of the problem. So we must take into account the following special feature of this verbal material.

- This verbal material consists in expressions which do not normally occur in the context of these women's daily lives, because of a clear censorship on these issues for many reasons (avoid worrying the family, maintain an image of strength, avoid being rejected, etc.). Since the interviews were set up in a noncritical context, away from “social censorship,” these contents were revealed.
- The most interesting statements that give an account of the suffering of these women and the affective aspects of their experience did not appear when expected, for example, when asking them a direct question on some issue, cause of a defense mechanisms that led them to leave those experiences behind and go away from those in which they had felt vulnerable. But in the course of free speech, these experiences were reported when the defense vanished, what justified our method. We will quote some of them here textually.
- While the investigation progressed, both the women and the researchers benefited from the verbal material in two ways: first the analysis of women experiences became more and more concise and detailed since the verbal expressions helped them to name, put in order, and clarify their experiences which were initially confused enough to overwhelm them. Miller [16] describes this phenomenon as “significantization” and letting the women felt relaxed and less stressed. Second, the interviewer was able to obtain a much higher quality speech in describing women feelings.
- The interviewed women were able to bring to mind circumstances that had been apparently forgotten but showed by the emotional vivacity deployed in the interviews. The verbalization of many of these facts was accompanied by honest emotional expressions, making the doctor's office a suitable context for the emotional relief (catharsis).

As we stated in a previous work, you must consider classifying the patients speech into “useful statements” and “useless statements” [17]. The latter are generally related to stereotypes (to make a good impression, avoid giving an image of weakness, repeat sentences heard before in a social context, etc.). For this reason we had to make specific tactical interviews in which women were reminded that they were not going to be judged or criticised at no time and that they could express themselves openly and unrestrictedly, making sincere verbalizations that had a greater interest for us.

 We are not using quantitative methods to deal with these patients (scales, surveys, questionnaires, etc.). We do not think it is appropriate for the following reasons: (1st) questionnaire works as a distraction, since it forces the person to focus on it instead of her own particular feelings losing both the time and the verbal material that give an account of the problems, (2nd) the experiences to be reported do have a qualitative rather than quantitative value [18, 19], and (3rd) what you get from clinical scales (anxiety, depression, obsessions, etc.) is often what is expected without reaching a new knowledge (e.g., it is obvious that women with breast cancer may suffer from anxiety, what is easily demonstrable with high score in anxiety scale). Therefore, we consider that the use of these methods is a difficulty more than a help, in this type of research [17].

## 3. Results

In the early stages of the disease acute emotional reactions are produced due to the women's attempt to adapt themselves to the new situation [20]. Later, when the surgical wounds heal and acute experiences are relieved, specific attitudes of the mastectomised women emerge, being more organised and having firm positions. These are developed in a gradual way and are related to the experiences of the body which has been altered by the ablation. On the following paragraphs we will give an account of these assertions.

### 3.1. The Fracture of the “Imaginary Body”

The breast is part of the female “body image,” being appreciated from the erogenous (alo-/autoerotic) point of view, which is often the expression of her own worth and power. Such limits may be seen in the interviews, in whose space many different retrospective attitudes about the image of this anatomic area (she is proud of her breast, she hides or disguises it, wants to show them in order to receive a praise, makes her feel confident, it is related to shame, etc.) are recorded. Hence, we have included it in the Sexual Body Schema (SBS) (14.15) not just for producing erotic sensations but for the value the woman gives it and, eventually, gives to herself because of its possession.

Mastectomy, as a breast removal, involves the loss of this worthy image, which provokes the fracture of the “corporal imaginary” (a discontinuity in the SBS), which is not observed in the surgical treatment of other female tumours. Therefore, after surgery, there is a fall in value (or erotic value) in two ways.

(a)   In relation to herself, the loss of the breast is experienced by women as an attack to the body image worrying about aesthetic features from that moment, which provokes that she does not feel beautiful: “When I see myself, I do not feel I have any charm, and this is a huge problem for me. I try to accept it, but I cannot.” Then it is not strange that she avoids looking at the mirror, which is a reference to the personal charm that no longer exists: “My appearance was like a circus clown, I felt sorry for myself,” and also avoids all those situations where she has to expose her body to the gaze of others (beaches, pools, gyms, etc.), in a way to hide herself, what we take as a “nonexhibitionist” attitude: “I used to walk naked around the house, but when I was operated I did not do it anymore”; “I enjoyed going to the nudist colony, I took pleasure in the feeling of getting into the water and swim naked, now I'm ashamed of that and I've stopped doing it.” The latter is understood as a way of not wanting to face a “mirror” in other people, fearing to receive a negative image from them.

At the same time, the loss of courage related to a single part of the “body image” is transmitted to the complete self-image and also to the whole personality, showing then a characteristic chain of thought: “my breast is not worthy” → “my body is not worthy” → “I'm not worthy.” The following sentences of patients testify this: “I used to see positive things on me, but not now, it seems I'm discovering more and more imperfections in my body”; “I'm no longer worthy as a person, I consider myself a complete failure”; “The truth is that I'm no longer good for anything, I'm a wreck.” We identify a generalization here which expands to the “whole” person, having internal connections established between different groups of representations in the same organization of the ego [21].

This leads to a fall in the self-esteem (“non-self-esteemed” feelings) that drive the woman not to like herself or even to reject herself, leading her to an attitude of introversion, inward-looking, shyness, insecurity, confinement, and/or social inhibition, which did not exist before the problem or at least were not so emphasised. We can even talk about feelings of inferiority: “I hate the way I am, because I do not feel like the other women, I'm not complete,” “I feel I'm not worthy and I do not know how to explain it,” “When I'm with my friends I become a shy person since I feel I'm inferior”; “I do not understand how this can make such an influence on me, since it not only stop me in intimacy with my sentimental couple, but also with the visitors who come home and I'm speechless when I'm with them, as though I had no words and could not express myself. This had not happened to me before.” Such feelings can be compensated by developing activities that previously were lack of interest, studying, working, reading, embellishing, and so forth: “I've started to study and I feel better now. I did not think I could do it, but here I am, the disease has brought me something good.” Many of these women find in these reactions a way to evade themselves and avoid thinking about their problems. More disturbing was those patients who had “reaction formations” which brought them to appear egoist and arrogant: “I've faced all this with a lot of integrity and I have proved to myself many times that I can deal with any problem;” in these circumstances having a subsequent collapse is not uncommon [22].

(b) In relation to others, what happens to the woman herself is reflected in her surrounding environment; that is, her body appearance may now be described through the manifestations, statements, or opinions from those around her, especially her couple, family, or friends, so she is more sensitive to any sign of rejection or disdain, perceiving any verbal or gestural expression of others as an affront. This makes her vigilant and show, quite often, a little bit dysphoric or irritating reactions.

These women try to hide the loss of a breast, having to deal with filling a bra or prosthesis since they are afraid others may discover their physical absence and also they see any glance as an attack to a zealously guarded privacy. They feel uncomfortable with people's curiosity about their physical condition and, especially, if they look insistently at the lost breast, feeling easily intimidated. Exaggerated interpretations abound here, and it is not easy to determine what is true and what is a figment of her imagination when they perceive rejection in place of esteem in gestures of others.

It is not uncommon in this context, the presence of phobic symptoms associated with social contact [23, 24]. Then they experience an extreme fear to rejection, which may lead them to refuse to return to their jobs after the period of recovery. Some of them kept in secret both the disease and surgery, and it was difficult for them to show their feelings: “I have not told anyone what happened to me. My husband, my mother and my daughters were the only ones who knew about my problem.” In such a case, the living circumstances are a matter of shame and women show a tendency to stay at home, having no desire to go to organizations or support groups, due to the fact that others can find out about what happens: “I suffer from low self-esteem and I spend more time at home.” Isolation and inactivity contribute to the problem, as they have more time to think about the situation of disability and the problems with people around them, what increased false interpretations of simple comments.

In some cases we recorded non-delusional self-references when they went out with the thought that they were being observed or they thought others saw them as an “oddball.” It is as if the other (magically) could detect the missing breast with his eyes despite having the body covered: “When I went out I felt everyone was looking at me, I felt naked in front of a people's jury,” “It seemed like my prosthesis moved constantly and everybody was waiting for the next move.”

For the mastectomised woman, the relationship with her partner is essential. In fact the latter is involved in her own thoughts, so that if she does not value herself neither does her partner: “If I hate myself, how do you think I'll be likeable to my husband?” Hence, they are quite sensitive in the relationship, and their partners do not know how to treat them and are afraid to do something to disturb, damage, or influence them in some way. These women are alert over the behaviour of men and are afraid of being rejected; this happens even after leaving the hospital: “I was afraid of not being able to attract him and that complicated everything. Then, he reacted very well and he did not give importance to it at all, as if nothing had changed”; “The fact of not having a breast made me lose spontaneity when I was with my husband.” They can even interpret as a sign of rejection or disdain the fact that he does not take the lead in the physical contacts.

For these reasons, it is reasonable that these women have a loss of sexual desire, often suffering a deterioration in their relationships; this can be related to the fact that they are ashamed of their damaged body image: “Seeing myself like that, ugly! I spent a long time without having sexual relationships.” Most women were reluctant to show their partner the surgical wound: “I had sex with my husband wearing a shirt and a bra. I could not allow him to see me so. I did not like me.”

In case of single women they were afraid of meeting a man and the idea of telling him about their problem specially at the moment of facing her naked body: “If I met someone else, how could I say I've lost a breast? How and when could I be undressed in front of him? It's a problem.” 

The “corporal imaginary” can also be broken by other changes which take place after the surgery and which are related to the absence of the breast, such as the following: the swelling of the arm caused by the lymphedema, which provokes brachial asymmetry: “it was a living hell for me to look at myself in the mirror and see my swollen arm and the lack of my breast," and the alopecia that follows the chemotherapy, may be even more feared than the removal of the breast because it is impossible to hide. However, we found women who are shaved and did not mind showing bald pates or they faced the problem with more serenity after thinking about it for a while. The capillary prosthesis gives no ideal solution because it also caused problem in their relationship with frequent self-references: “People looked at me when I went down the street, I could not stand it and came back to my home quickly”; “I felt people had changed their perception of me”; “I thought they had realised that I was wearing a wig, because when I was talking they did not look into my eyes, but to my forehead.” In addition, wigs cause a special fear at the prospect of flying away or moving, the binomial intervening concealment/unveiling (to the gaze of others). The loss of eyebrows and eyelashes make the problem more difficult because they think her face is losing its definition.

(c) Increase in weight and the swelling as a result of the inactivity due to the fatigue and fluid retention as an after-effect of medicines, also produces a rejection of the new image.

### 3.2. The Problem of “Femininity” in the Mastectomised Woman

We have witnessed how the patients associate the lack of the breast with a loss of “femininity” without knowing what is going on exactly. We verified how their discursive approaches to the problem were clearly expressive of discomfort: “I looked at my breast and I thought: you're not such a woman”; “It takes your femininity away. People say: The important thing is you overcome the disease, but I said to the doctor “please, do not remove my breast””; “I stopped feeling like a woman, something had disappeared. I needed to have something in that part.” 

There is a constant loss of desire in these women, often attributed to the treatments (e.g., hormonal), but we detected in the interviews that there were numerous aspects which had to do with their “femininity” so after recognize this issue her desire improved on. 

We determined that this decrease in the libido is related to the fact that the woman does not feel themselves to be attractive once the breast is removed (being bald, with no eyebrows, with no eyelashes, and more weight). She is defaced, she has stopped being beautiful, and she does not feel enough capacity for seducing: “How can you think we may have a relationship when I'm such a frump?”

We refer here to something which is essential for women as a part of the “femininity”: the issue of “desirability,” meaning the fact of whether she is desired or not. The desire does not work in the same way in the “female position” and the “male” one because in case of a woman the basic principle of being desired/desirable is needed: “if I'm desirable, then I'll feel desire” or “I desire him because he desires me.”

These psychological events are altered in the mastectomised woman because, if she does not think her body is attractive, it will be difficult for her to be able to provoke the desire in a man: “I do not think I look pretty anymore and that's why I have no desire to be with him”; “Something was missing on me, I had lost all my sensuality”; “I always felt very proud of my breast, I used to go out without a bra. It was horrible when I had my breast removed. At first I could not believe it and then I did not feel pretty, I thought it was impossible any man liked to be with me, not even my own partner”; “The breast is very important for men, I do not know if it has to do with culture or education. When men meet a woman they use to look at her breast.”

They may even feel, in association with the other gender, that they are in a position of deception or fake: “I was walking down the street and several men were looking at my breast, I thought I was wearing the prosthesis. A man looks at two boobs as it's feminine”; “They look at you but you know they're looking something inexistent; it's a lie and you feel bad.”

Despite that their sentimental partners do not often show a lack of desire for them and still consider them attractive, the machinery seems to be broken: “I know perfectly well that he supports me and he says his feelings has not changed, but I cannot believe it, really.”

Therefore, “being female” means to many women paying a special attention to their body (to take care of themselves, to dress themselves up, to get themselves ready, etc.) to make it desirable, and also involving a man in all that process to make him “a prey of their many charms.” In this sense, it is thought that to lose a breast is the equivalent to lose a part of their “femininity,” and, what is more, some of them feel like they lose their own identity: “I said, I'm not the same anymore”; “With this, doctors steal your own personality.”

We must add that they also have to change their clothes so as to be able to hide the lack of their breast or the prosthesis, so they cannot use freely low-cut neck clothes, tight or transparent: “I had to find another type of clothing that was very different from my previous style. It's very hard”, “I did not feel feminine because I had to wear a bra which looked like my grandmother's. Mastectomy limits yourself when wearing your underwear.” 

This change of clothing prevents them from seducing by showing their body shapes, including the cleavage or the intermammary sulcus, both of them being a powerful incentive to attract the gaze (and the desire) of a man: “I feel I'm a less flirtatious woman because I have always loved to wear low-cut neck clothes and miniskirts.”

Interestingly when the breast reconstruction has been successful and they were satisfied with the results, they started to feel feminine again: “Now I have that shape, I know that's not my breast, but I can already dress as a female and I do not have to wear an orthopaedic bra”; “I have an expander and it has provoked a turn of the screw in my life.” “I have more desire to wear a low-cut neck shirt or to crouch at any time. Before the reconstruction when I crouched I saw a hole. I am much happier.” As a matter of fact, many authors have agreed in asserting that one of the reasons to have a breast reconstruction is to have the freedom to use different types of clothes [25–31].

### 3.3. Mutilation or “the Real” of the Body

Although the operation took place some time ago, many patients still had in their minds the issue of mutilation. This one belongs not to the “body image” (already discussed) but to a psychological dimension which Lacan [32] called “the Real” of the body to refer to a hole impossible to be integrated into the psyche. Indeed, any organic disease or corporal disorder must be integrated into the psyche through a process of symbolization or “significantization” [16] to keep mental stability.

The phenomena related to this dimension are shown from the early stages of the operation, although they can decrease, they never disappear completely. In the early stages we observed the fear of the patients when they looked at the mirror or when they touched that part of their body, especially when they were cleaning them up, doing it in a special way (with their eyes closed, in the dark, etc.) since they could not face the postoperation modifications. This is a way of refusing to be aware of the mutilation which is considerd as a defense from “the Real.”

 What we have here is the horror or the fear of the impressive presence of a deformed body, on which it is impossible to articulate a word, which makes the woman feel speechless. That is the reason why the modern medicine tries to avoid, by all means, this terrible event, by developing ways to reconstruct the breast so that its absence is not exposed: “It was a great relief for me when I checked my breasts were still in their place after the operation. I thanked God because that awful situation had already gone.”

Some women may avoid this confrontation during a long time and when they finally face it they receive a strong impact: “I was not prepared to perceive the scar in the mirror until several weeks after the surgery. Although my doctor had explained to me what he had done during the operation and, when I did it, I was paralyzed by shock. There was a missing part on me”; “The image I found out in the mirror caused me a great disappointment, I could not see it. I thought life was very hard for women,” “I looked at myself in the mirror and started to cry”; “It took me six months to look at myself in the mirror, although I saw many images on the Internet. At the end I was able do it and I cannot explain how I felt.”

It is not uncommon to display depersonalization experiences: “I did not know who I was, I did not recognize myself,” “I thought I'd never be the same anymore. I felt weird,” “it was an enigma to me where I was or who I was.”

All these experiences often turn into sexual refuse, “asexuality,” or “aversion to sex.” 

We must not ignore that some secondary effects of the treatments may also be unacceptable beyond the mere aesthetics (e.g., The swelling of the arm can cause many limitations, chemotherapy causes nausea and fatigue, etc.)

### 3.4. Particularly Problematic Cases

The psychological elaboration of mastectomised woman is not always performed favourably, and we have already mentioned that her personality is essential concerning this matter [20]. Therefore, those who have more difficulties and who may be more affected by the disease are those who dedicate too much time and energy to take care of their body in order to preserve their physical appearance. They are also people who avoid enlightening other longer-lasting qualities than the physical ones. An intense dedication to the body care takes away a valuable time which could be used for other activities, thereby losing the opportunity to acquire representational material from other sources.

We refer to women who (a) take all their self-esteem from corporal values as well as physical attractiveness, while they seek and live looking for the attention of those who live around them, (b) describe their breast as a very important factor of the sexual desire (desirability), and, above all, (c) have too high expectation of ideal body. It seems clear that an uncompleted body image is produced in mastectomy and a woman with these (hysteriform) characteristics is unable to assimilate since it differs dramatically from what we might call the “ideal sexualised body schema” (ISBS).

## 4. Argument and Conclusion

In the preceding pages we have strived to study the women affected by breast cancer, a field in which advances are becoming larger and larger every day, so that the early detection, the change in treatment guidelines, and the possibility of reconstruction have changed the outline of this medical issue. But the particular circumstances of each case provoke that not all the affected women can benefit from breast reconstruction techniques keeping a normal body appearance. Many of them lose the breast, what leads them to psychological problems [8, 10, 11, 33, 34]. So they should be supported by a mental health professional skilled in these matters.

Once the mastectomy has been produced, the woman suffers too much due to the physical change she has experienced, although this psychological pain can sometimes be hidden behind another such as the fear of disease and its possible reappearance, but this does not mean it is not present. Halsted, the founder of breast surgery, focused on the issue of saving the lives of the patients, leaving aside the “body image.” In this sense he wrote the following: “The disability is irrelevant in comparison to the life of the patient. In addition, these patients are old, they have an average of nearly 55 years.” These claims are now meaningless.

At first, the mastectomised woman shows a fracture in the “corporal imaginary,” that is, in the mental image of the body that everyone has, which is directly linked to intersubjectivity. Then, the self-image depends on how the others perceive it and vice versa. These interactions were already suspected by Sartre [35] and developed by Schilder [36] and Lacan [37], being the latter who related them to narcissism. As a consequence, the “body image” is inextricably linked to the self-appraisal.

The breast, in this situation, constitutes itself an essential erotic part that gives value to the woman. It is understandable that the removal of such a worthy part for her leads to the idea of stopping be desirable. This is something that affects, in a greater or lesser extent, all the women who pass through this situation. They avoid, from that moment, any situation which reveals its “imaginary incompleteness,” hiding it from other people and also from themselves. The lack of value of a body part, representationally, is widespread to the rest of the person. It is then that the fall of self-esteem takes place leading to introversion and social inhibition. The feeling of inferiority has a rather unique development here, since it breaks with the particular competition which leads them to think “I'm better than you”; now, by contrast, that cannot be said anymore. In that moment we witness the Adlerian compensations, which need not be considered pathological but an excess of the opposite may occur, in which case the woman may show her arrogance and immoderation: “I can deal with everything,” “nothing can stop me,” and so forth.

This broken “corporal imaginary” is given, mostly, in the emotional-sexual field, as it is in privacy, where they are fully examined. The possibility of rejection here is felt in depth. Hence, they become very sensitive to the behaviour of their couples. But these, taking our experience as an example, tend to be careful and keep on behaving as they used to do despite the change. They declare frequently that they do not mind the consequences the operation has had on their bodies. It is not weird that some surgeons, presuming the magnitude of the problem, recommend their patients to have sex as soon as possible. 

It is not strange that mastectomy discusses the matter of “femininity” and it should be treated adequately, as almost all the interviewed women understand the removal as an attack to their femininity. In order to understand the problem, it is necessary to get into the field of “masculinity-femininity” and “the games of attraction between men and women.” We discovered then that the “femininity” or “the female position” is associated with the disposition of the body of the woman based on the attractiveness it has for a man. Its function is to become attractive as well as awaken the desire of him, an aspect which we have named “desirability” in this paper. This phenomenon does not occur when there is a missing breast and she feels disfigured, and the femininity weakens. The comfort that comes when they see their couples, tireless and faithful, do not seem to have lost the desire, while they are expecting to detect any kind of reluctance on them.

Obviously, a series of behaviours are intended to praise and enhance the physical through the mending and clothing in order to get the “desirability.” Logically thinking, the clothing worn by the mastectomised woman does not necessarily tie with such behaviour, by altering the position of the women. The interviewed women complain that doctors “do not put in the shoes of the women” and that may be true in some cases, since it is advisable to scrutinize the experiences we have described to do it.

The quality of the previous sentimental relationship clearly influences the existing communication, particularly, this entire journey, so that, if it is a good relationship, we will consider it a positive case. We have seen that, in the frequent periods of high emotion, if the communication leaves a lot to be desired, any act or omission is received with much more power and impact.

One aspect that we do not want to leave out, perhaps one of the most dramatic, is the one that has to do with the problem of mutilation, which lies in “the Real” of the body [32]. It is something impossible to integrate into the psychic life and that occurs in the hospital in many ways, being the most common is to avoid a direct conflict with the mirror or any other situation which shows them that “body fracture.” It is common that, in later times, a part of the phenomenon still remains (e.g., feeling disgust, repulsion, or shivers). 

For all the explained reasons, not only the woman has to make a physical effort to recover from a sick body, which often leaves her exhausted, but also she must make an extra mental work to prepare herself to all the adverse events described above. These are not always easy to assume, especially in the cases where the patient depends too much on her body, uses her image in her job, has a lack of independency since she only exists when other people look and/or admire her, and she is at the beck and call of powerful “physical ideals” that make her think in achieving a “perfect body” impossible to reach.

Therefore, the patient has to try to fight herself as Baudrillard insists in one of his famous texts that “The body has become the most beautiful object of consumption,” to develop new personal skills that lead the woman to another state with which she can feel less disconcerted. 
